# Lead Exposure and Cardiovascular Disease among Young and Middle-Aged Adults

**DOI:** 10.3390/medsci7110103

**Published:** 2019-11-06

**Authors:** Emmanuel Obeng-Gyasi

**Affiliations:** Environmental Health and Safety Program, Department of Built Environment, North Carolina Agricultural & Technical State University, Greensboro, NC 27411, USA; eobenggyasi@ncat.edu; Tel.: +1-336-285-3132

**Keywords:** lead exposure, lead young adult, lead middle-aged adult, lead cardiovascular

## Abstract

Lead and its effects on cardiovascular-related markers were explored in this cross-sectional study of young adults (18–44 years) and middle-aged adults (45–65 years) from the United States using the National Health and Nutrition Examination Survey (NHANES), 2009–2016. Degrees of exposure were created using blood lead level (BLL) as the biomarker of exposure based on the epidemiologically relevant threshold of BLL > 5 μg/dL. The mean values, in addition to the percentages of people represented for the markers of interest (systolic blood pressure [SBP], diastolic blood pressure [DBP], gamma-glutamyl transferase [GGT], non-high-density lipoprotein cholesterol [non-HDL-C]) were explored. Among those exposed to lead, the likelihood of elevated clinical markers (as defined by clinically relevant thresholds of above normal) were examined using binary logistic regression. In exploring exposure at the 5 μg/dL levels, there were significant differences in all the mean variables of interest between young and middle-aged adults. The binary logistic regression showed young and middle-aged adults exposed to lead were significantly more likely to have elevated markers (apart from DBP). In all, lead affects cardiovascular-related markers in young and middle-aged U.S. adults and thus we must continue to monitor lead exposure to promote health.

## 1. Introduction

Lead exposure begins as early as pregnancy and can affect those exposed throughout their life as it persists in the blood and the bone [1,2]. Exposure to lead may occur in many settings via water, air, soil, dust, and food [3,4,5,6] and may subsequently induce toxic pathology in many organ systems within the human body [7,8,9,10].

Cardiovascular diseases are the primary cause of mortality in the world [11]. Cardiovascular-related markers include systolic blood pressure (SBP), for which a value of less than or equal to 120 mm Hg is considered normal. An SBP reading of 130 mm Hg or more is medically classified as stage I hypertension. Another marker of interest is diastolic blood pressure (DBP); clinically, a normal DBP must be less than 80 mm Hg. Values greater than or equal to 140 mm Hg for SBP is considered stage II hypertension, while values greater than or equal to 90 mm Hg is considered stage II for DBP. These guidelines by the American College of Cardiology (ACC) and the American Heart Association (AHA) recently replaced guidelines which had higher medical thresholds to reach these health classifications [12]. Lead exposure and its relation to hypertension have been demonstrated in the literature [8,12,13], with increases in blood lead level (BLL) increasing blood pressure [14,15,16].

Non-high-density lipoprotein cholesterol (non-HDL-C), a measure of low-density lipoprotein (LDL) cholesterol and very low-density lipoprotein (VLDL) cholesterol, predicts heart disease risk better than markers such as LDL cholesterol [17]. A non-HDL-C value > 160 mg/dL is considered elevated [18,19]. The mechanisms by which lead alters cholesterol synthesis is still being studied, but it is potentially involved in promoting enzymes essential for cholesterol synthesis (enzymes such as 3-hydroxy-3-methyglutaryl coenzyme A [HMG CoA] reductase, farnesyl diphosphate synthase, squalene synthase, and lanosterol 14a-demethylase CYP51) while also inhibiting enzymes involved in its breakdown (e.g., 7α-hydroxylase) [20].

Gamma-glutamyl transferase (GGT) is an enzyme that is present in hepatocytes, biliary epithelial cells, renal tubules, and the pancreas and intestine. GGT is a microsomal enzyme, and toxicants, drugs, or dietary alterations can induce its activity. It is also a sensitive marker for oxidative stress [21,22], a condition in which free reactive oxygen species (ROS) and free radicals cause potential biological damage [23].

Lead’s effect on the cardiovascular system and cardiovascular-related markers has been well noted in the literature [24]. The mechanism by which lead induces hypertension may be related to oxidative stress, inflammation, alterations in the renin–angiotensin–aldosterone system, alteration of vasoactive and volume regulatory hormones, and nitric oxide dysregulation, among other mechanisms [24].

No level of lead exposure is safe, but exposure above the 5 μg/dL level has been widely accepted to be elevated in adults [25]. Age is also a key factor in lead exposure, as longer exposure results in worse health outcomes [26,27]. It is important to examine how the cardiovascular system of young adults and middle-aged adults are differentially affected by exposure to lead. To this effect, key cardiovascular-related markers, such as non-HDL-C, GGT, SBP, and DBP, can serve to inform about lead’s effect among adults exposed at all levels and above the threshold considered elevated.

It is also key then to examine potential markers or mechanisms which may result in worse cardiovascular health. This study examines the effects of lead on the abovementioned cardiovascular-related markers in young adults and middle-aged adults, looking at both the associations between lead and these markers and the likelihood of elevated marker levels among those exposed to lead.

## 2. Methods

### 2.1. Study Hypothesis

This study hypothesized that lead exposure is associated with adverse alterations in oxidative stress (GGT), heart disease risk (non-HDL-C), and blood pressure (SBP and DBP) in middle-aged adults more so than younger adults, as they may have been exposed over a longer duration of time, thereby adversely altering their cardiovascular health. In addition, this study hypothesized that lead would be significantly associated with more adverse cardiovascular-related markers in middle-aged adults as compared to younger adults. The objective in this study was to investigate the effects of lead exposure by analyzing non-HDL-C, GGT, SBP, and DBP in young (18–44 years) and middle-aged (45–65 years) U.S. adults.

### 2.2. Research Design

The relationships between lead and SBP, DBP, GGT, and non-HDL-C were explored using the National Health and Nutrition Examination Survey (NHANES) 2009–2016, a representative sample of the noninstitutionalized U.S. population. Four sets of two-year cycle data were combined to build the dataset. Data from young adults and middle-aged adults were analyzed using guidelines provided by the 2009–2016 NHANES tutorial [9].

Metal assays were conducted using whole blood samples for the NHANES 2009–2016 at the National Center for Environmental Health (NCEH). Inductively coupled plasma mass spectrometry (ICP-MS) was used to measure BLLs with the lower limit being 0.07 µg/L. Biochemistry markers were measured using a Beckman Synchron LX20 and Beckman UniCel^®^ DxC800 Synchron (Collaborative Laboratory Services), including a Roche Modular P chemistry analyzer (University of Minnesota, Minneapolis, MN, USA). The data analysis was performed in Stata SE/15.0 (StataCorp, College Station, TX, USA), which permitted for the adjustment of sample weights, strata, and clusters in the complex design.

### 2.3. Statistical and Analytical Approaches

The data from young and middle-aged adults were analyzed for the cardiovascular-related markers in this cross-sectional study. The level of elevated exposure was created using the BLL based on the epidemiologically relevant threshold of 5 μg/dL. The mean values, in addition to the representative percentages for the markers of interest, were explored at the 5 μg/dL exposure level.

The likelihood of elevated clinical markers as defined by clinically relevant thresholds of exposure (SBP > 120 mm Hg and DBP > 80 mm Hg for blood pressure, GGT above the median of the dataset (18 U/L) for oxidative stress, and non-HDL-C > 160 mg/dL for heart disease risk) were explored via binary logistic regression. The clinical markers served as the dependent variables, while the independent variable was the blood lead levels.

In this study, individual models were used to explore each exposure–outcome variable in logistic regression. The data were adjusted for different combinations of gender, body mass index (BMI), income, ethnicity, alcohol consumption, and smoking based on the literature [28,29,30,31,32]. Where relevant with the complex design, weights were adjusted to ensure the analysis was representative of the noninstitutionalized U.S. general adult population. A Shapiro–Wilk test revealed that BLL was not normally distributed, so it was natural log transformed. A *p*-value < 0.05 determined significance while a value of *p* < 0.10 was deemed moderately significant.

## 3. Results

### 3.1. Clinical Markers of Interest

The clinical markers of interest in the young and middle-aged adults were analyzed to determine the mean values of the markers of interest among the two groups of adults. BLLs were more elevated in middle-aged adults than in younger adults, with SBP also following the same pattern. Further, concerning DBP, non-HDL-C, and GGT, higher values were found in the middle-aged adult group. Regarding BLL, SBP, DBP, non-HDL-C, and GGT there was a significant difference between young adults and middle-aged adults (*p* < 0.001). Table 1 summarizes these results.

### 3.2. Clinical Markers at Exposure Levels of BLL above 5 μg/dL

Mean levels of the clinical markers of interest among the adults in the two age groups exposed to BLLs above the 5 μg/dL threshold were explored. At the BLL of >5 μg/dL, SBP was more elevated as the age group increased. DBP, non-HDL-C, and GGT increased from young adulthood to middle age. At the BLL of >5 μg/dL, regarding DBP, there was a significant difference between young adults and middle-aged adults (*p* = 0.003).

For SBP at the 5 μg/dL level and above, there was a significant difference between young adults and middle-aged adults (*p* < 0.001). Regarding non-HDL-C, at the 5 μg/dL level, there were no significant differences. Finally, for GGT, there were no statistically significant differences at the 5 μg/dL level. The results can be found in Table 2 below.

### 3.3. Percentage of Adults with Markers above Exposure Levels of BLL >5 μg/dL

The data was explored at clinically relevant thresholds of SBP > 120 mmHg and DBP > 80 mmHg for blood pressure, GGT above the median (18 U/L) for oxidative stress, and non-HDL-C > 160 mg/dL for heart disease risk. Data for the percentage of adults above the thresholds for each marker was computed. At the 5 μg/dL level, the percentage of adults above the threshold for SBP increased with increasing age group. For DBP, non-HDL-C, and GGT, there was an increase from young adulthood to middle-age.

For SBP at the at the 5 μg/dL level, there was a significant difference between young adults and middle-aged adults (*p* < 0.001). For DBP at the 5 μg/dL level, there was a statistically significant difference between young adults and middle-aged adults (*p* = 0.029). For non-HDL-C, at the 5 μg/dL level, there was a statistically significant difference between young adults and middle-aged adults (*p* = 0.002).

At the 5 μg/dL and above level for GGT, there was a statistically significant difference between young adults and middle-aged adults (*p* = 0.006). Results for the percentage of adults above the thresholds for each marker are shown in Table 3 below.

### 3.4. Likelihood of Elevated Clinical Markers by Age Group

The likelihood of elevated clinical markers as defined by clinically relevant thresholds of exposure (SBP > 120 mmHg and DBP > 80 mm Hg for blood pressure, GGT above the median (18 U/L) for oxidative stress, and non-HDL-C > 160 mg/dL for heart disease risk) were explored via binary logistic regression. The clinical markers served as the dependent variables with the independent variable being the natural log transformation of blood lead levels. This was explored among young and middle-aged adults. Results can be found in Table 4 below:

## 4. Discussion

Lead has a profound effect on cardiovascular health. Low-level lead exposure affects the public’s health and cardiovascular health in adults [28,33]. In the United States, exposure occurs due to the legacy of lead in mediums such as water, paint, and soil, thereby keeping populations continuously exposed [34,35,36]. Among adults in the U.S., the workplace is the primary location where exposure occurs [33].

In this study examining young and middle-aged adults from the U.S., it was hypothesized that BLLs would be higher for middle-aged adults, as they were more likely to have endured a longer duration of exposure. The mean BLL was found to be higher in middle-aged adults than younger adults in this study. This potentially indicates that a longer duration of exposure has left this group with elevated BLLs, which has put them at risk of diseases such as cardiovascular disease. It is key to note that since BLL is a measure of mainly acute exposure with a half-life of 30 days in blood [37], it is a non-perfect measure.

Lead generally showed a trend of increased association with higher SBP in this study as age increased. With age comes increasing arterial stiffness [38], which plays a significant role higher blood pressure during aging. Studies of metals which examine the mechanisms of their involvement in cardiovascular pathology suggest that lead may potentially play a role in arterial stiffness [24]. This was suggested when comparing young adults to middle-aged adults in this study.

DBP showed a similar trend to SBP, although its association (as an elevated marker) was not significant in middle-aged adults—as it was for SBP. This may be due to different pathophysiological mechanisms for SBP and DBP [39]. Indeed, pathophysiological alterations in the arterial wall make older people more prone to conditions such as isolated systolic hypertension, as changes including endothelial dysfunction and elastin calcifications increase the risk [40]. Finally, previous studies have found, in both men and women, that increases in blood lead concentration are associated with an elevation in SBP and DBP [16].

Regarding cholesterol, studies have demonstrated that lead potentially plays a role in cholesterol dysfunction [8,41]. The mean value of non-HDL-C in this study was larger in middle-aged adults compared to young adults. In a study by MacLean and colleagues, non-HDL-C increased with age in men until around 54 when it peaked, while in women it increased more gradually until age 54 when it increased appreciably to exceed the values of men [34]. In this study, the odds of those with elevated non-HDL-C having high BLL was true for young and middle-aged adults.

The mean GGT level was larger in middle-aged adults compared to young adults. At all exposure levels, GGT generally did not vary much between the two age groups. Lee and co-authors found associations between blood lead and GGT in adults [42]. Serum GGT has also been associated with all-cause cardiovascular disease in older adults [43].

Among the adults, increasing levels of lead exposure generally pushed those individuals above the threshold for elevated clinical markers for the variables of interest. This potentially speaks to the role lead may play in many multifactorial diseases as—along with genetics, diet, and other factors—environmental exposure to lead may contribute to people being propelled toward various cardiovascular pathologies. Thus, it is very important to mitigate exposure to promote public health.

Potential adverse health outcomes in young and middle-aged adults occurred both above and below the epidemiological threshold of 5 μg/dL. This potentially confirms that no level of lead is safe and that more must be done to mitigate or eliminate sources of lead exposure.

### Mitigation of Exposure

While being aware of the fact that many of the cardiovascular diseases resulting partly from lead exposure are multifactorial, comprehensive approaches must be explored to mitigate exposure to protect public health. Adult exposure to lead occurs mainly in the workplace; thus, interventions to lessen the effects of lead exposure may begin there and must factor in the hierarchy of controls [44]. Mitigating other sources of exposure may include reducing lead levels in gasoline, paint, plumbing pipes, and food cans [6]. In addition, technological solutions to reduce lead emissions from smelting facilities can decrease the risk on populations, as can soil remediation and behavioral interventions such as hand washing [6]. Regarding health, chelation treatment, which reduces the body burden of those with high BLLs, is an effective means to reduce risk [6].

A limitation of the study is the fact that measurement of BLLs represents short-term rather than longer-term exposure, as lead has a half-life of roughly 30 days in blood. Another limitation is the cross-sectional design, which makes it difficult to determine the temporal sequence of exposure and effect. Finally, a larger dataset would have allowed for examining older adults, who have presumably accumulated exposure over a longer period of time, but the amount of data for this group was too small to produce statistically reliable estimates for detailed demographic sub-domains.

## 5. Conclusions

Cardiovascular diseases are an issue of significant public health concern. Lead exposure affects cardiovascular markers in young and middle-aged adults, with higher exposure (with increasing age) generally resulting in worse health outcomes. Efforts must continue to stop exposure as early as pregnancy to prevent lead from accumulating and later affecting individuals into adulthood.

## Figures and Tables

**Table 1:** Variables of interest among categories of adults.

Variables	N Young Adults	Young Adults (SE)	N Middle-Aged Adults	Middle-Aged Adults (SE)
BLL—μg/dL (SE)	7730	1.03 (0.026)	5744	1.62 (0.044)
SBP—mmHg (SE)	9757	115.27 (0.201)	7119	124.15 (0.351)
DBP—mmHg (SE)	9757	68.99 (0.292)	7119	72.99 (0.262)
non-HDL-C—mg/dL (SE)	10,165	132.74 (0.636)	7512	149.21 (0.761)
GGT—U/L (SE)	10,130	24.02 (0.378)	7489	30.87 (0.723)

**Table 2:** How variables manifested at an exposure level of 5 μg/dL and above among adults.

Variables at 5 μg/dL and above	N Total	Young Adults (SE)	Middle-Aged Adults (SE)
SBP—mmHg (SE)	379	116.47 (1.65)	132.19 (2.50)
DBP—mmHg (SE)	379	70.16 (1.65)	76.22 (1.66)
non-HDL-C—mg/dL (SE)	408	149.18 (5.28)	155.17 (8.26)
GGT—U/L (SE)	396	37.59 (6.46)	39.91 (4.65)

**Table 3:** Percentage of adults above the clinical threshold for the markers of interest.

Variables at 5 μg/dL	N Total	Young Adults (SE)	Middle-Aged Adults (SE)
SBP—mmHg (SE)	379	34.39 (6.51)	64.68 (4.93)
DBP—mmHg (SE)	379	13.32 (5.71)	29.11 (5.15)
non-HDL-C—mg/dL (SE)	408	68.23 (6.40)	90.33 (2.41)
GGT—U/L (SE)	396	62.24 (6.98)	82.71 (3.85)

**Table 4:** Logistic regression of markers of interest among adults.

Variable of Interest	Young Adults (18–44 Years) Adj. Odds Ratio (95% CI)	*p*-Value	Middle-Aged Adults (45–65 Years) Adj. Odds Ratio (95% CI)	*p*-Value
SBP **	1.21 (1.07–1.38)	0.003	1.32 (1.14–1.52)	<0.001
DBP **	1.32 (1.10–1.58)	0.003	1.16 (0.98–1.38)	0.076
non-HDL-C *	1.58 (1.45–1.72)	<0.001	1.50 (1.31–1.71)	<0.001
GGT ^+^	1.55 (1.39–1.72)	<0.001	1.37 (1.15–1.64)	0.001

** adjusted for gender, body mass index (BMI), income, ethnicity, alcohol consumption, and smoking; * adjusted for gender, BMI, income, and ethnicity; ^+^ adjusted for gender, BMI, income, ethnicity, and alcohol consumption.
